# Natural language processing diagnosed behavioural disturbance phenotypes in the intensive care unit: characteristics, prevalence, trajectory, treatment, and outcomes

**DOI:** 10.1186/s13054-023-04695-0

**Published:** 2023-11-04

**Authors:** Marcus Young, Natasha E. Holmes, Kartik Kishore, Sobia Amjad, Michele Gaca, Ary Serpa Neto, Michael C. Reade, Rinaldo Bellomo

**Affiliations:** 1 Data Analytics Research and Evaluation (DARE) Centre, Austin Health and The University of Melbourne, Heidelberg, VIC Australia; 2 School of Computing and Information Systems, The University of Melbourne, Parkville, Melbourne, VIC Australia; 3 Australian and New Zealand Intensive Care Research Centre, School of Public Health and Preventive Medicine, Monash University, Melbourne, Australia; 4 Department of Intensive Care, Austin Hospital, 145 Studley Rd, Heidelberg, Melbourne, Australia; 5 Department of Critical Care, School of Medicine, The University of Melbourne, Parkville, Melbourne, VIC Australia; 6 Department of Intensive Care, Royal Melbourne Hospital, Melbourne, Australia; 7 Department of Infectious Diseases, Peter Doherty Institute for Infection and Immunity, University of Melbourne, Victoria, 3000 Australia; 8 Faculty of Medicine, University of Queensland, Brisbane, QLD Australia; 9 Joint Health Command, Australian Defence Force, Brisbane, QLD Australia; 10 Department of Intensive Care Medicine, Royal Brisbane and Women’s Hospital, Brisbane, QLD Australia

**Keywords:** Intensive care, Delirium, Critical illness, Agitation, Mortality, Antipsychotic drugs

## Abstract

**Background:**

Natural language processing (NLP) may help evaluate the characteristics, prevalence, trajectory, treatment, and outcomes of behavioural disturbance phenotypes in critically ill patients.

**Methods:**

We obtained electronic clinical notes, demographic information, outcomes, and treatment data from three medical-surgical ICUs. Using NLP, we screened for behavioural disturbance phenotypes based on words suggestive of an agitated state, a non-agitated state, or a combination of both.

**Results:**

We studied 2931 patients. Of these, 225 (7.7%) were NLP-Dx-BD positive for the agitated phenotype, 544 (18.6%) for the non-agitated phenotype and 667 (22.7%) for the combined phenotype. Patients with these phenotypes carried multiple clinical baseline differences. On time-dependent multivariable analysis to compensate for immortal time bias and after adjustment for key outcome predictors, agitated phenotype patients were more likely to receive antipsychotic medications (odds ratio [OR] 1.84, 1.35–2.51, *p* < 0.001) compared to non-agitated phenotype patients but not compared to combined phenotype patients (OR 1.27, 0.86–1.89, *p* = 0.229). Moreover, agitated phenotype patients were more likely to die than other phenotypes patients (OR 1.57, 1.10–2.25, *p* = 0.012 vs non-agitated phenotype; OR 4.61, 2.14–9.90, *p* < 0.001 vs. combined phenotype). This association was strongest in patients receiving mechanical ventilation when compared with the combined phenotype (OR 7.03, 2.07–23.79, *p* = 0.002). A similar increased risk was also seen for patients with the non-agitated phenotype compared with the combined phenotype (OR 6.10, 1.80–20.64, *p* = 0.004).

**Conclusions:**

NLP-Dx-BD screening enabled identification of three behavioural disturbance phenotypes with different characteristics, prevalence, trajectory, treatment, and outcome. Such phenotype identification appears relevant to prognostication and trial design.

**Graphical abstract:**

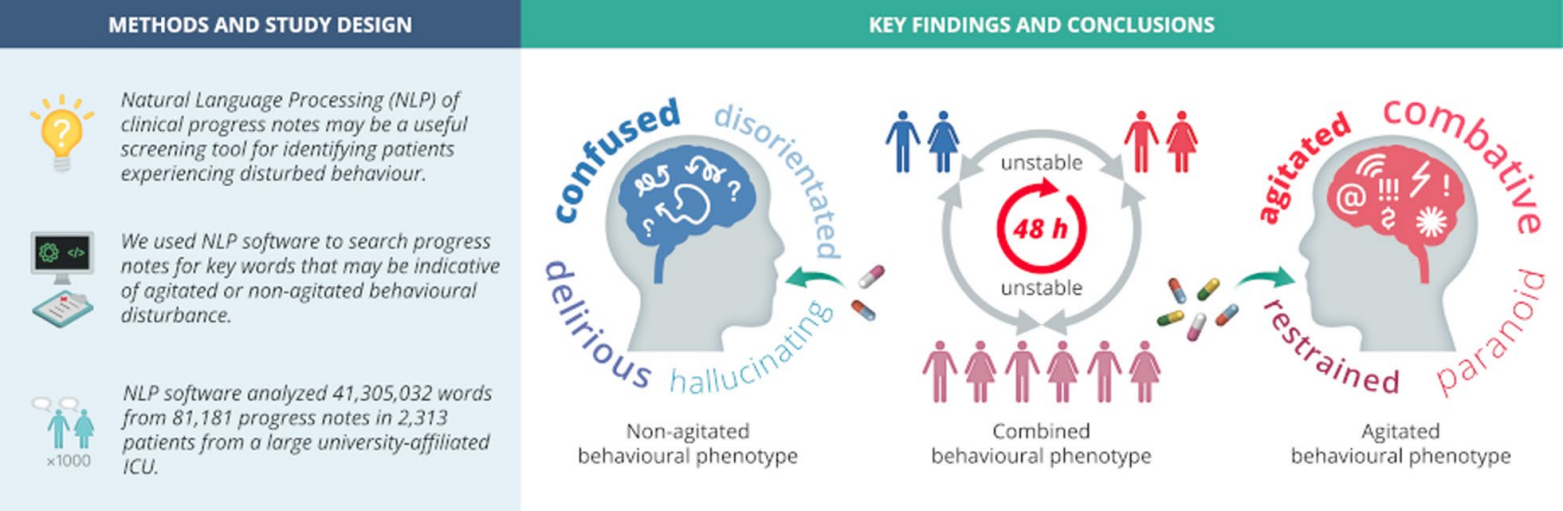

**Supplementary Information:**

The online version contains supplementary material available at 10.1186/s13054-023-04695-0.

## Background

The study of the epidemiology of disturbed behavioural phenotypes in critically ill patients is both novel and challenging [1]. In part, this is because such phenotypes are multifaceted manifestations of a poorly understood underlying neurocognitive state. Thus, they cannot be measured and can only be described by words. Moreover, the frequently cited reference for their description [2] is itself an aggregation of words, provides limited guidance for the systematic identification of behavioural phenotypes, and was not intended for use in the intensive care unit (ICU). Furthermore, the widely used screening tools for cognitive and behavioural dysfunction in critically ill patients, the Confusion Assessment Method for the Intensive Care Unit (CAM-ICU) [3] and the Intensive Care Delirium Screening Checklist (ICDSC) [4] only include limited guidance for the identification of behavioural phenotypes.

Notwithstanding the above difficulties, using CAM-ICU and ICDSC, several studies have reported that phenotypes of delirium do exist and may be associated with different morbidity and mortality rates [5–7].

Such studies have also suggested that there might be clinical value in further investigating the characteristics, prevalence, trajectory, treatment, and outcomes of disturbed behavioural phenotypes, a similar but not identical concept to delirium [8–14]. In this regard, natural language processing (NLP) of caregivers’ notes has recently emerged as a screening tool for behaviour in critically ill patients [8–14]. NLP has also been recently used to describe the syndrome of NLP-diagnosed behaviour disturbance (NLP-Dx-BD) [15]. Different from delirium screening tools, the identification of this syndrome requires the application of NLP to screen caregiver notes for the purpose of detecting words or phrases that describe disturbed behaviour (see “Methods” section). Such NLP-Dx-BD is a condition observed in critically ill patients and has high sensitivity for the identification of patients who later go on to be treated with antipsychotic medications in the ICU [8].

Moreover, although NLP can be used to read caregiver’s clinical notes for the purpose of identifying words and phrases such as “combative” or “confused”, these words may also be indicative of different phenotypes of NLP-Dx-BD. Thus, “combative” implies an agitated phenotype, while, in the absence of words to indicate psychomotor agitation, “confused” implies a non-agitated phenotype, and, finally, the presence of both phenotypes within a defined reporting period (e.g., 24 h) implies a combined phenotype.

We hypothesised that NLP could be used to detect words and phrases associated with distinct behavioural phenotypes and that such NLP-defined phenotypes would be associated with clinically important differences in other patient characteristics. Accordingly, we used NLP to study the characteristics, prevalence, trajectory, treatment, and outcomes of phenotypes of NLP-Dx-BD in a cohort of critically ill patients.

## Methods

### Study design

We performed a non-interventional, retrospective study of a cohort of critically ill adult patients (≥ 18 years old) admitted to the three ICUs of a university affiliated hospital in Melbourne, Australia between 1 May 2019 and 31 December 2020. The study was approved by the Austin Hospital Human Research Ethics Committee (LNR/19/Austin/38), which waived the requirement for informed consent. For patients with multiple admissions, only the first was included. No other exclusion criteria were applied. During the study period, all patients received care designed to reduce the risk of developing delirium including family visits, dimmed lights at night, minimal interaction to facilitate night-time sleep, and the use of visual and auditory aids as required.

### Data collection and manipulation

We obtained the patient clinical progress notes entered into the electronic health record (EHR) by doctors, nurses, physiotherapists, and other allied health professionals. We analysed these notes using NLP techniques. As previously described [8, 15], each progress note was converted into sentence vectors, tokenised and searched for the presence of words indicative of agitated or non-agitated behaviour (Natural Language Toolkit; NLTK 3.5) [16], a process equivalent to the first step of Large Language Model Generative Pre-trained Transformer strategies recently popularised by OpenAI.

The words used in our NLP model were derived from the results of a previously published survey of clinical staff who were asked to identify words, terms or expressions that they would associate with disturbed behaviour and possible delirium [17].

In this study, we further categorised each of these words as being suggestive of agitated or a non-agitated behavioural disturbance state, while the presence of negation or resolution words was also determined (Table 1). Accordingly, all notes for the same patient were aggregated and categorised into four groups of NLP diagnosed behavioural disturbance (NLP-Ex-BD): (1) agitated, when only agitated words were present in available notes; (2) non-agitated, when only non-agitated words were present in available notes; (3) combined, when both agitated and non-agitated words were present in available notes; and (4) no disturbance, when no agitated or non-agitated words were present in any note available.

**Table 1:** Words suggestive of behavioral disturbance

Agitated	Agitated	Agitation	Aggression	Aggressive
	Combative	Endangering	Paranoia	Paranoid
	Restrained	Restraint	Shackled	Violence
	Uncooperative	Violent		
Non-agitated	Confused	Confusion	Delirium	Delirious
	Disorientation	Disorganized	Disorganised	Disorientated
	Distraction	Disturbed	Delusion	Fluctuating
	Inattention	Incoherent		
Negation	No	Not	Nill	Nil
Resolution	Resolved	Resolving	Cleared	Clearing
	Ceased			

We use the term NLP-Dx-BD instead of delirium for the sake of accuracy. Importantly, we wish to emphasise that the words in Table 1 are intuitively associated with cognitive deterioration, which is recognised with delirium as well. However, we are also aware that we do not have sufficient data, for example, to exclude the fact that agitated behaviour was not, in fact, due (in a very few individuals) to underlying severe pain and/or dementia.

Baseline and outcome data were obtained from the Australian and New Zealand Intensive Care Society Adult ICU Patient Database run by the Centre for Outcome and Resource Evaluation [18]. Data detailing the use of antipsychotic medications were obtained from the hospital’s electronic medication management system.

### Exposure

The primary exposure of this study for a given patient was the occurrence at any time of having a word/s indicating agitated or non-agitated behaviour, or combined behavioural disturbance, within the notes recorded during their ICU stay.

### Outcomes

The primary outcome of this study was the use of antipsychotic medications.

Although such treatment is controversial [19–22], antipsychotic medication use was chosen as the primary outcome measure because we were studying behaviour and considered it likely that such medications would be used differently according to the presence or absence of an agitated behavioural phenotype [23, 24].

Secondary outcomes included ICU and hospital mortality and 28-day mortality censored at hospital discharge as well as ICU and hospital length of stay and duration of mechanical ventilation.

### Statistical analysis

All continuous data are reported as median (quartile 25%–quartile 75%) and categorical data as numbers and percentage. Baseline, clinical characteristics and outcomes of the patients were compared among the groups using the Fisher exact test and Kruskal–Wallis test.

Multivariable logistic regression models were used to assess the impact of the exposure on the use of antipsychotic medications and hospital clinical outcomes, according to each phenotype. The model was adjusted by age, type of admission, and by the Australian and New Zealand (ANZ) Risk of Death (ANZROD) after log transformation [25]. ANZROD is a local recalibration of the APACHE III score which adjusts for the persistent lower than expected mortality in ANZ and contains variables such as admission diagnosis and pre-existing comorbidities [25]. As previously demonstrated, ANZROD is an accurate outcome predictor and explains most of the mortality found in ICUs in Australia and New Zealand [26]. Effect estimates were reported as odds ratio (OR) with its 95% confidence interval (CI). To account for immortal time bias, we conducted a time-dependent Cox proportional hazard model for the primary outcome and hospital mortality that considered all measurements available in each note. For the primary outcome, only exposure variables happening before the first outcome (first time the patient received an antipsychotic) were included in the model to avoid exposures measured after the medication had been given. Due to pairwise comparisons, the significance level was adjusted using a Bonferroni method and a *p* < 0.01 was considered statistically significant. All analyses were case complete analyses and were conducted in R v.4.0.2 (R Foundation, Vienna, Austria) [27]

## Results

### Characteristics and prevalence of NLP-Dx-BD phenotypes

We studied 2931 patients, 79,807 progress notes, and 17,110,747 words. Among the study patients, 1436 (49.0%) were Natural Language Processing Diagnosed Behavioural Disturbance (NLP-Dx-BD) positive. Of the positive patients, 225 (15.7% of positive and 7.7% of all patients) were categorised into the agitated phenotype; 544 (38.0% of positive and 18.6% of all patients) into the non-agitated phenotype and 667 (46.4% of positive and 22.7% of all patients) into the combined phenotype (Additional file 1: Fig. S1). Patients categorised into the combined phenotype displayed the characteristics of the agitated and non-agitated phenotypes at the same or different times during their admission.

The words ‘agitated’, ‘agitation’ and ‘restraint’ were the most common words detected from the NLP-Dx-BD agitated word list (Additional file 1: Fig. S2a) and ‘delirium’, ‘confused’ and ‘fluctuating’ the most common words from the non-agitated word list (Additional file 1: Fig. S2b).

The baseline (first 24 h of ICU admission) characteristics of the study patients are summarised in Table 2, which shows multiple significant differences according to the presence of NLP-Dx-BD and of its three different phenotypes. Compared to patients with the agitated phenotype, those with the non-agitated phenotype were older, had higher APACHE III scores, different admission diagnoses, and higher serum creatinine levels. However, they were also less likely to receive invasive ventilation and vasopressor drugs.

**Table 2:** Baseline characteristics of the included patients

	No disturbance (*n* = 1495)	Agitated (*n* = 225)	Non-agitated (*n* = 544)	Combined (*n* = 667)	*p* value
Age, years	63.1 (51.1–73.2)	55.2 (42.3–67.3)	68.1 (56.6–78.0)	63.5 (49.4–74.3)	< 0.001
Male gender—no. (%)	873 (58.6)	146 (65.2)	332 (61.0)	413 (61.9)	0.175
Body mass index (kg/m^2^)	28.3 (24.5–33.2)	27.9 (24.4–32.0)	27.5 (22.8–32.3)	27.1 (23.2–31.8)	0.369
APACHE III	44 (33–58)	50 (34–66)	57 (43–71)	61 (46–77)	< 0.001
ANZROD	1.8 (0.6–5.7)	3.0 (0.9–9.1)	4.5 (1.4–16.1)	7.0 (2.2–20.3)	< 0.001
Type of admission—no. (%)					< 0.001
Medical	709 (47.4)	144 (64.0)	328 (60.3)	447 (67.0)	
Elective surgery	460 (30.8)	34 (15.1)	108 (19.9)	83 (12.4)	
Urgency surgery	326 (21.8)	47 (20.9)	108 (19.9)	137 (20.5)	
Planned admission—no. (%)	514 (34.4)	46 (20.4)	121 (22.2)	112 (16.8)	< 0.001
MET call admission—no. (%)	244 (16.3)	37 (16.4)	121 (22.2)	137 (20.5)	0.007
Cardiac arrest—no. (%)	24 (1.6)	7 (3.1)	7 (1.3)	22 (3.3)	0.025
Acute renal failure—no. (%)	16 (1.1)	8 (3.6)	11 (2.0)	35 (5.3)	< 0.001
Admission diagnosis—no. (%)					< 0.001
Cardiovascular	486 (32.5)	59 (26.2)	136 (25.0)	155 (23.2)	
Gastrointestinal	258 (17.3)	30 (13.3)	93 (17.1)	119 (17.8)	
Gynaecological	6 (0.4)	1 (0.4)	0 (0.0)	1 (0.1)	
Haematological	22 (1.5)	0 (0.0)	6 (1.1)	2 (0.3)	
Metabolic	91 (6.1)	29 (12.9)	25 (4.6)	62 (9.3)	
Musculoskeletal	47 (3.1)	0 (0.0)	8 (1.5)	13 (1.9)	
Neurological	84 (5.6)	21 (9.3)	64 (11.8)	102 (15.3)	
Renal and genitourinary	98 (6.6)	6 (2.7)	29 (5.3)	20 (3.0)	
Respiratory	219 (14.6)	45 (20.0)	69 (12.7)	100 (15.0)	
Sepsis	145 (9.7)	28 (12.4)	90 (16.5)	62 (9.3)	
Trauma	39 (2.6)	6 (2.7)	24 (4.4)	31 (4.6)	
ICU source of admission—no. (%)					< 0.001
Emergency department	362 (24.2)	86 (38.2)	151 (27.8)	214 (32.1)	
Operating room	778 (52.0)	81 (36.0)	215 (39.5)	218 (32.7)	
Other hospital (not ICU)	119 (8.0)	17 (7.6)	49 (9.0)	65 (9.7)	
Other hospital ICU	13 (0.9)	4 (1.8)	7 (1.3)	32 (4.8)	
Ward	221 (14.8)	35 (15.6)	120 (22.1)	136 (20.4)	
Other	2 (0.1)	2 (0.9)	2 (0.4)	2 (0.2)	
Co-existing disorders—no. (%)					
Diabetes	320 (21.4)	37 (16.4)	122 (22.4)	144 (21.6)	0.298
Chronic lung disease	190 (12.7)	25 (11.1)	82 (15.1)	82 (12.3)	0.380
Chronic cardiovascular disease	68 (4.5)	7 (3.1)	31 (5.7)	39 (5.8)	0.273
Cirrhosis	88 (5.9)	14 (6.2)	60 (11.0)	79 (11.8)	< 0.001
Chronic kidney disease	157 (10.5)	24 (10.7)	85 (15.6)	81 (12.1)	0.019
Chronic immune disease	14 (0.9)	2 (0.9)	4 (0.7)	11 (1.6)	0.435
Immunosuppression	141 (9.4)	22 (9.8)	63 (11.6)	58 (8.7)	0.382
Liver failure	11 (0.7)	4 (1.8)	5 (0.9)	7 (1.0)	0.408
Lymphoma	14 (0.9)	2 (0.9)	14 (2.6)	2 (0.3)	0.003
Metastatic cancer	78 (5.2)	10 (4.4)	22 (4.0)	17 (2.5)	0.037
Leukemia	39 (2.6)	4 (1.8)	15 (2.8)	16 (2.4)	0.908
Organ support—no. (%)					
ECMO	2 (0.1)	0 (0.0)	1 (0.2)	6 (0.9)	0.039
Vasopressor or inotropes	588 (39.7)	130 (57.8)	248 (46.0)	447 (67.6)	< 0.001
Invasive ventilation	544 (36.7)	151 (67.1)	216 (40.1)	448 (67.7)	< 0.001
Non-invasive ventilation	87 (5.9)	14 (6.2)	40 (7.4)	58 (8.8)	0.093
Renal replacement therapy	32 (2.2)	15 (6.7)	32 (5.9)	109 (16.5)	< 0.001
Laboratory tests					
pH	7.39 (7.35–7.43)	7.38 (7.33–7.43)	7.40 (7.35–7.44)	7.38 (7.32–7.44)	0.010
PaO_2_/FiO_2_	319 (243–395)	307 (210–376)	301 (223–372)	264 (164–357)	< 0.001
PaCO_2_ (mmHg)	40 (35–45)	40 (35–45)	39 (35–44)	40 (35–46)	0.153
Lactate (mmol/L)	1.9 (1.3–2.8)	2.4 (1.6–3.7)	2.2 (1.6–3.3)	2.4 (1.7–3.7)	< 0.001
Highest creatinine (µmol/L)	85.0 (67.0–122.0)	83.0 (65.5–121.0)	103.0 (74.8–158.2)	101.0 (68.0–167.0)	< 0.001
Lowest platelet, × 10^9^/L	184 (131–253)	181 (134–258)	171 (116–246)	173 (115–239)	0.001
Vital signs					
Lowest MAP (mmHg)	66 (61–73)	66 (59–71)	64 (58–72)	64 (59–70)	< 0.001
Highest RR (breaths/min)	20 (18–25)	20 (17–26)	22 (18–28)	20 (17–25)	< 0.001
Highest temperature (°C)	37.2 (36.7–37.5)	37.1 (36.6–37.6)	37.2 (36.7–37.6)	37.3 (36.8–37.8)	0.002
Urine output (mL)	1527 (1080–2100)	1570 (1142–2184)	1480 (1055–2053)	1490 (1010–2175)	0.193

Data are median (IQR) or *N* (%)

*APACHE* acute physiology and chronic health evaluation, *MET* medical emergency team, *ICU* intensive care unit, *ECMO* extracorporeal membrane oxygenation, *MAP* mean arterial pressure, *RR* respiratory rate

Compared with agitated phenotype patients, those with a combined phenotype were older, had higher APACHE III scores, a different source of admission, and were more likely to receive vasopressor drugs and renal replacement therapy. Finally, compared with the non-agitated phenotype those with the combined phenotype were younger, had higher APACHE III scores, different types of admission, were more likely to have acute kidney injury, had different admission diagnoses and source of admission, and were more likely to receive mechanical ventilation, vasopressor drugs and renal replacement therapy. A pairwise comparison of these three different phenotypes is presented in Additional file 1: Table S1, which confirmed the findings of Table 2 and identified the relationship between phenotypes.

### Early trajectory of NLP-Dx-BD phenotypes

**Fig. 1:**
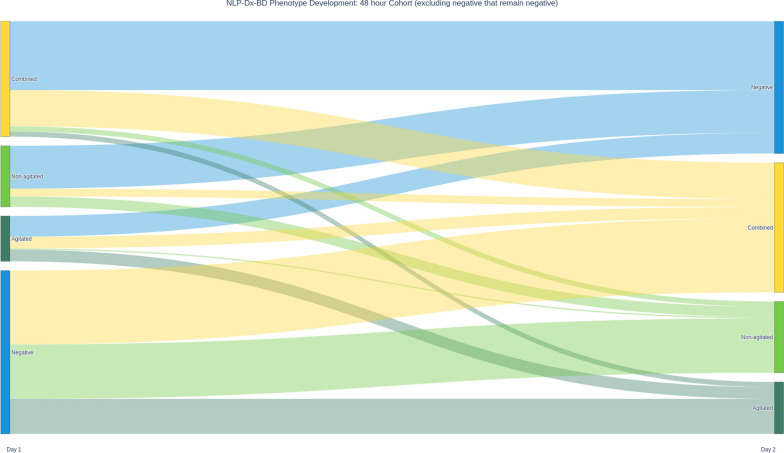
Alluvial plot illustrating the changes in NLP-Dx-BD phenotype over time in patients who were positive for the condition at some stage in the first 48 h. Most patients transitioned from one phenotype to another or achieved resolution indicating that not only is NLP-Dx-BD a potentially fluctuating condition but that phenotypes are similarly fluctuating

Most episodes of NLP-Dx-BD occurred in the first 2 days (Additional file 1: Fig. S3). Thus, we studied the trajectory of NLP-Dx-BD phenotypes within these first 48 h among patients who, on day one or day two, had developed NLP-Dx-BD (Fig. 1, Additional file 1: Table S6). We found that behavioural phenotypes within this population changed dynamically.

More than half (59.55%) of the patients diagnosed with the combined phenotype of NLP-Dx-BD on the day of admission were negative (resolution) for NLP-Dx-BD by the second day. Approximately a third (31.46%) continued to display the combined phenotype and a small number transitioned to either the agitated (4.49%) or non-agitated (4.49%) phenotype. Patients with the non-agitated phenotype on the day of admission were similarly likely to achieve resolution of their NLP-Dx-BD (70.21%). However, approximately one third (17.02%) remained in a non-agitated phenotype state with a smaller number (12.77%) transitioning to the combined phenotype and no patients in the non-agitated phenotype transitioned to an agitated phenotype on day two. Finally, almost a half (45.72%) of the patients with the agitated phenotype on admission achieved resolution of NLP-Dx-BD, approximately one in two (51.42%) transitioned to the combined phenotype or remained agitated and only one (2.86%) became non-agitated.

### Primary outcome: antipsychotic medication use

The raw numbers for each antipsychotic drug used in each phenotype are presented in Table 3, which shows significant differences in their use according to phenotype. In the unadjusted pairwise comparison (Additional file 1: Table S2), antipsychotic drug use was significantly different only when the combined phenotype was the comparator. Thus, their use was significantly greater in the combined vs both other phenotypes (*p* < 0.001). This was also true for each individual agent (*p* < 0.001).

**Table 3:** Antipsychotic drug use for study patients according to phenotype

	No disturbance (*n* = 1495)	Agitated (*n* = 225)	Non-agitated (*n* = 544)	Combined (*n* = 667)	*p* value
Medications—no. (%)					
Any APD	31 (2.1)	16 (7.1)	36 (6.6)	174 (26.1)	< 0.001
Haloperidol	1 (0.1)	2 (0.9)	3 (0.6)	76 (11.4)	< 0.001
Olanzapine	5 (0.3)	5 (2.2)	6 (1.1)	34 (5.1)	< 0.001
Quetiapine	11 (0.7)	8 (3.6)	17 (3.1)	140 (21.0)	< 0.001
Risperidone	1 (0.1)	0 (0.0)	1 (0.2)	3 (0.4)	0.190

Data are median (IQR) or *N* (%)

*APD* antipsychotic drugs

When analysing the use of antipsychotic drugs as outcome and considering all patients and groups as time-dependent variables (Additional file 1: Table S3), the univariable model showed that the odds ratio for receiving such drugs was greatest for the agitated phenotype. This was confirmed on multivariable modelling considering each phenotype as time dependent variable (Table 4) and was true for invasively ventilated and non-ventilated patients. Moreover, compared with patients without disturbance, the impact of NLP-Dx-BD with an agitated component (agitated or combined) on the prescription of anti-psychotic medications was strong (Additional file 1: Table S4).

**Table 4:** Multivariable models with use of anti-psychotic drugs as outcome and according to the use of invasive ventilation and considering the phenotypes as time-dependent variable

	All patients		Invasive ventilation		No invasive ventilation	
	Odds ratio (95% CI)	*p* value	Odds ratio (95% CI)	*p* value	Odds ratio (95% CI)	*p* value
Group						
Agitated versus non-agitated	1.84 (1.35–2.51)	< 0.001	1.52 (1.06–2.18)	0.023	1.86 (0.96–3.57)	0.064
Agitated versus combined	1.27 (0.86–1.89)	0.229	1.11 (0.70–1.78)	0.648	1.63 (0.76–3.52)	0.209
Non-agitated versus combined	0.63 (0.42–0.94)	0.022	0.75 (0.46–1.23)	0.260	0.57 (0.29–1.12)	0.105

All information from all notes available were included as time-dependent variables

### Secondary outcomes

The raw data for the secondary outcomes of the study patients are shown in Additional file 1: Table S4 and the unadjusted pairwise comparison is presented in Additional file 1: Table S5. The duration of ventilation, ICU length of stay and hospital length of stay were significantly greater for the combined phenotype vs. the agitated and non-agitated phenotype groups (*p* < 0.001). The unadjusted ICU mortality, hospital mortality and 28-day mortality rates were not significantly different across the phenotypes. However, once multivariable models were applied with hospital mortality as outcome and groups as time-dependent variables (Table 5), patients with the agitated phenotype were more likely to die than patients with other phenotypes (OR 1.57, 1.10–2.25, *p* = 0.012 vs. non-agitated phenotype; OR 4.61, 2.14–9.90, *p* < 0.001 vs. combined phenotype). This association was strongest in those patients receiving mechanical ventilation when compared with the combined phenotype (OR 7.03, 2.07–23.79, *p* = 0.002). A similar increased risk was also seen for patients with the non-agitated phenotype compared with the combined phenotype (OR 6.10, 1.80–20.64, *p* = 0.004).

**Table 5:** Multivariable models with hospital mortality as outcome and considering the groups as time-dependent variable

	All patients		Mechanical ventilation		No mechanical ventilation	
	Odds ratio (95% CI)	*p* value	Odds ratio (95% CI)	*p* value	Odds ratio (95% CI)	*p* value
Group						
Agitated versus non-agitated	1.57 (1.10–2.25)	0.012	1.34 (0.89–2.02)	0.159	2.08 (0.99–4.34)	0.052
Agitated versus combined	4.61 (2.14–9.90)	< 0.001	7.03 (2.07–23.79)	0.002	3.70 (1.18–11.58)	0.024
Non-agitated versus combined	2.31 (1.13–4.73)	0.021	6.10 (1.80–20.64)	0.004	0.99 (0.40–2.47)	0.989

All information from all notes available were included as time-dependent variables

## Discussion

### Key findings

We applied Natural Language Processing (NLP) to evaluate the characteristics, prevalence, trajectory, treatment, and outcomes of behavioural disturbance phenotypes in critically ill patients. We identified three major phenotypes: agitated, non-agitated, and combined. We found that each of these three phenotypes was associated with different patient characteristics. Of these, the combined phenotype was the most common, followed by the agitated phenotype. However, the trajectory of patients was such that movement from one phenotype to another in the first 48 h was common (65.66%). Moreover, during this time, it was uncommon for non-agitated patients to develop an agitated phenotype, many agitated patients achieved resolution of their NLP-Dx-BD, and both non-agitated and agitated phenotype patients typically transitioned to a combined phenotype. We also found significant differences in the use of antipsychotic medication. Thus, in time-variant multivariable models and after adjustment, patients with an agitated component to their NLP-Dx-BD were significantly more likely to receive antipsychotic medications overall compared with those patients with non-agitated NLP-Dx-BD. Finally, we found that patients with the combined phenotype had longer unadjusted duration of invasive ventilation, ICU stay, and hospital stay and greater mortality. However, once multivariable models were applied with hospital mortality as the outcome and groups as time-dependent variables, it was patients with the agitated phenotype who were more likely to die than patients with other phenotypes, especially among those patients receiving mechanical ventilation.

### Relationship to previous studies

#### Characterisation

Behavioural disturbance phenotypes based on NLP analysis of caregiver notes have not been previously defined. However in a parallel fashion, three phenotypes of delirium have been previously described: hyperactive, hypoactive and mixed [28–31]. Although there is no gold standard for the identification of these phenotypes, the linguistic constructs and psychomotor descriptors used in previous studies appear broadly consistent with the categorisation of the NLP-Dx-BD search terms used in our study [32]. Nonetheless, NLP-Dx-BD phenotypes are focussed on unstructured continuous observation of behaviour by care givers and their relationship with structured intermittent screening tool-based assessment is unknown [3, 33].

#### Prevalence

The prevalence of the phenotypes of NLP-Dx-BD phenotypes in critically ill patients is unknown and ours is the first study to explore this concept. However, studies using structured intermittent screening tools reported that between 0.3 and 45.9% of patients displayed agitation, 0.5–91% displayed a non-agitated state and 1–69.5% displayed both [7, 20, 22]. The prevalence of the NLP-Dx-BD phenotypes identified in our study falls within these ranges.

#### Trajectory

To our knowledge, the trajectory of behavioural phenotypes of critically ill patients has not been reported. Moreover, studies of the epidemiology and treatment of delirium using intermittent structured screening tool-based assessments have not reported on their dynamic nature, thus implying stability [34, 35]. In contrast, and for the first time, we have demonstrated in detail that behavioural phenotypes, as identified by NLP, are unstable within the first 48 h of critical care admission.

### Treatment and outcomes

Antipsychotic medications remain widely used for the treatment of disturbed behaviour in critically ill patients [36, 37] with 70% of antipsychotic medication used in acute care prescribed for its treatment [38]. Further, several studies have reported higher rates of administration for agitated patients [23, 39]. In our study, we also found antipsychotic medication use was significantly more likely in the combined NLP-Dx-BD group followed by the agitated groups (both groups having an agitated component). This implies that it is the presence of agitation that drives antipsychotic drugs prescription.

Using intermittently applied structured-assessment tools, a previous study of non-ventilated patients suggested that mortality did not differ between the agitated, non-agitated and combined states [40]. However, in our study, after appropriate adjustments and assessing the group as a time-dependent variable (an adjustment not applied to previous studies), ICU mortality was highest for the NLP-Dx-BD agitated group. This suggests that NLP assessment of continuous caregiver observation may identify a cohort of patients that differs from that identified by intermittently applied structured-assessment tools.

### Implications of study findings

Our findings imply that NLP-Dx-BD may be used to identify three clinically relevant phenotypes. Further, our findings suggest that the combined phenotype may be dominant. However, they also suggest that the early trajectory of these phenotypes is complex with dynamic changes from one phenotype to the other, indicating that such phenotypes are unstable. Moreover, on multivariable analysis and overall, patients with an agitated component to their NLP-Dx-BD (agitated phenotype or combined phenotype) appear significantly more likely to receive antipsychotic medications. These observations imply that future studies of pharmacologic intervention should primarily focus on patients who present an agitated component to their phenotype, either in isolation or in a combination (combined phenotype).

### Strengths and limitations

Our study has several strengths. It is the first to use NLP to study the prevalence of behavioural phenotypes in critically ill patients. It is also the first to describe the early trajectory of these phenotypes in critically ill patients and to demonstrate their dynamic and unstable nature. Moreover, to the best of our knowledge, our study presents the first detailed information of the use of antipsychotic medications according to phenotype, thus providing further evidence of the validity of the NLP-Dx-BD construct. Finally, with current software, as soon as a key word describing an agitated state is entered into the electronic notes, such entry can be used to trigger alerts to clinicians, thus facilitating randomisation into interventional trials.

We acknowledge several limitations. First, our study was undertaken in a large intensive care unit system involving three ICUs within a university affiliated tertiary hospital in a resource-rich country. Therefore, our findings may not apply to other intensive care units in low or middle-income countries or to other ICUs with a different approach to the management of disturbed behaviour. Moreover, patients were not assessed for the presence or absence of delirium by independent adjudication personnel. However, we investigated behavioural disturbances. This is different in focus, concept, and technique from the assessment of delirium [8]. Thus, we cannot comment of the relationship between our findings and their relevance to intermittently applied delirium screening tools. Future investigations of such relationship may be of interest. The use of medications in the treatment of behavioural disturbance in critically ill patients is controversial. However, antipsychotic medications remain an important tool for moderating behaviour, are a reasonable proxy for disturbed behaviour (the target of our investigation), and are widely used in critically ill patients [22, 41]. Finally, clinicians may be unfamiliar with NLP techniques, which might generate scepticisms about our findings. However, clinicians are the very generators of the words we used in our study; NLP-based technology is fast becoming accepted in response to the arrival of Large Language Model Generative Pre-trained Transformer strategies, and our study is the first step toward machine learning approaches to the behavioural management of critically ill patients.

## Conclusions

For the first time, we demonstrated that Natural Language Processing of electronic caregiver notes enables the identification and characterisation of NLP-Dx-BD phenotypes in critically ill patients. Moreover, we found that the combined phenotype was dominant and that, in the first 48 h, it was common for critically ill patients within the study cohort to transition between behavioural phenotypes of NLP-Dx-BD. Importantly, after adjustment, patients with the agitated phenotype either in isolation or within a combined phenotype were significantly more likely to receive antipsychotic medications. These findings have important implications for our understanding of the epidemiology of phenotypes of disturbed behaviour in critically ill patients and for trial design in this field.

### Supplementary Information

**Additional file 1:** Online Supplement.

## Data Availability

Will individual participant data be available (including data dictionaries)? Yes. What data in particular will be shared? Individual deidentified records. What Other documents will be available? Ethics approval. When will the data be available (start and end dates)? From 1 year after publication for a period of 1 year. With whom? Researchers with a study protocol approved by an institutional ethics review board. For what types of analysis? Additional observational statistical analysis. By what mechanism will data be made available? By submission of a research protocol to the corresponding author.
