# Supplementary material for: Natural language processing diagnosed behavioural disturbance phenotypes in the intensive care unit: characteristics, prevalence, trajectory, treatment, and outcomes
Source: Crit Care. 2023 Nov 4;27:425. doi: 10.1186/s13054-023-04695-0 (PMC10625294; doi:10.1186/s13054-023-04695-0)

**Natural Language Processing Diagnosed Behavioural Disturbance Phenotypes in the Intensive Care Unit: Characteristics, Prevalence, Trajectory, Treatment, and Outcomes**

ONLINE SUPPLEMENT

| **Table S1 – Significance of Difference in Baseline Characteristics of Included Patients According to Behavioural Phenotype** | | | |
| --- | --- | --- | --- |
|  | **Agitated vs.**  **Non-agitated** | **Agitated vs. Combined** | **Non-agitated vs. Combined** |
| Age, years | < 0.001 | < 0.001 | < 0.001 |
| Male gender - no. (%) | 0.288 | 0.425 | 0.767 |
| Body mass index, kg/m^2^ | 0.741 | 0.598 | 0.801 |
| APACHE III | < 0.001 | < 0.001 | 0.001 |
| ANZROD | 0.001 | < 0.001 | 0.001 |
| Type of admission - no. (%) | 0.307 | 0.538 | 0.002 |
| Planned admission - no. (%) | 0.631 | 0.226 | 0.019 |
| MET call admission - no. (%) | 0.078 | 0.206 | 0.481 |
| Cardiac arrest - no. (%) | 0.133 | 0.999 | 0.024 |
| Acute renal failure - no. (%) | 0.210 | 0.371 | 0.004 |
| Admission diagnosis - no. (%) | < 0.001 | 0.020 | < 0.001 |
| ICU source of admission - no. (%) | 0.034 | < 0.001 | < 0.001 |
| Co-existing disorders - no. (%) |  |  |  |
| Diabetes | 0.064 | 0.104 | 0.728 |
| Chronic lung disease | 0.170 | 0.722 | 0.177 |
| Chronic cardiovascular disease | 0.147 | 0.119 | 0.999 |
| Cirrhosis | 0.044 | 0.016 | 0.717 |
| Chronic kidney disease | 0.088 | 0.633 | 0.093 |
| Chronic immune disease | 0.999 | 0.534 | 0.195 |
| Immunosuppression | 0.528 | 0.593 | 0.102 |
| Liver failure | 0.296 | 0.483 | 0.999 |
| Lymphoma | 0.171 | 0.265 | 0.001 |
| Metastatic cancer | 0.843 | 0.176 | 0.190 |
| Organ support - no. (%) |  |  |  |
| ECM1e | 0.999 | 0.346 | 0.137 |
| Vasopressor or inotropes | 0.003 | 0.009 | < 0.001 |
| Invasive ventilation | < 0.001 | 0.870 | < 0.001 |
| Non-invasive ventilation | 0.643 | 0.260 | 0.458 |
| Renal replacement therapy | 0.741 | < 0.001 | < 0.001 |
| Laboratory tests |  |  |  |
| pH | 0.098 | 0.854 | 0.013 |
| PaCO_2_, mmHg | 0.167 | 0.855 | 0.034 |
| Lactate, mmol/L | 0.205 | 0.803 | 0.037 |
| Highest creatinine, µmol/L | < 0.001 | < 0.001 | 0.557 |
| Lowest platelet, x 10^9^/L | 0.079 | 0.042 | 0.780 |
| Vital signs |  |  |  |
| Lowest MAP, mmHg | 0.462 | 0.170 | 0.484 |
| Highest temperature, ºC | 0.204 | 0.013 | 0.110 |
| Urine output, mL | 0.049 | 0.083 | 0.869 |
| *APACHE: Acute Physiology and Chronic Health Evaluation; MET: medical emergency team; ICU: intensive care unit; ECMO: extracorporeal membrane oxygenation; MAP: mean arterial pressure; RR: respiratory rate; ANZROD: Australian and New Zealand Risk of Death* | | | |

| **Table S2 – Unadjusted Comparison of Individual Antipsychotic Use According to Behavioural Phenotype** | | | |
| --- | --- | --- | --- |
|  | **Agitated vs.**  **Non-agitated** | **Agitated vs. Combined** | **Non-agitated vs. Combined** |
| Any APM | 0.875 | < 0.001 | < 0.001 |
| Haloperidol | 0.633 | < 0.001 | < 0.001 |
| Olanzapine | 0.314 | 0.088 | < 0.001 |
| Quetiapine | 0.824 | < 0.001 | < 0.001 |
| Risperidone | 0.999 | 0.576 | 0.632 |
| *APM: Antipsychotic Medication* | | | |

| **Table S3 - Univariable Models with Use of Anti-Psychotic Drugs as Outcome Considering Behavioural Phenotypes as Time-Dependent Variable* and According to the Use of Invasive Ventilation and** | | | | | | |
| --- | --- | --- | --- | --- | --- | --- |
|  | **All Patients** | | **Invasive Ventilation** | | **No Invasive Ventilation** | |
|  | **Odds Ratio**  **(95% CI)** | ***p* value** | **Odds Ratio**  **(95% CI)** | ***p* value** | **Odds Ratio**  **(95% CI)** | ***p* value** |
| Group |  |  |  |  |  |  |
| Agitated vs. Non-agitated | 1.82 (1.35 to 2.47) | < 0.001 | 1.53 (1.07 to 2.19) | 0.019 | 1.90 (1.03 to 3.49) | 0.039 |
| Agitated vs. Combined | 1.28 (0.87 to 1.89) | 0.207 | 1.19 (0.75 to 1.90) | 0.453 | 1.52 (0.74 to 3.14) | 0.257 |
| Non-agitated vs. Combined | 0.64 (0.43 to 0.94) | 0.025 | 0.78 (0.48 to 1.27) | 0.312 | 0.57 (0.29 to 1.12) | 0.102 |
| * All information from all notes available were included as time-dependent variables | | | | | | |

| **Table S4 - Multivariable Models with Use of Anti-Psychotic as Outcome Considering Behavioral Phenotypes as Time-Dependent Variables and According to Mechanical Ventilation with Inclusion of No Behavioural Disturbance Patients as Reference*** | | | | | | |
| --- | --- | --- | --- | --- | --- | --- |
|  | **All Patients** | | **Mechanical Ventilation** | | **No Mechanical Ventilation** | |
|  | **Odds Ratio**  **(95% CI)** | ***p* value** | **Odds Ratio**  **(95% CI)** | ***p* value** | **Odds Ratio**  **(95% CI)** | ***p* value** |
| **Group** |  |  |  |  |  |  |
| **No disturbance** | 1 (Reference) |  | 1 (Reference) |  | 1 (Reference) |  |
| **Agitated** | 7.46 (5.21 to 10.68) | < 0.001 | 5.62 (3.63 to 8.69) | < 0.001 | 8.76 (4.34 to 17.66) | < 0.001 |
| **Non-agitated** | 2.65 (1.76 to 4.01) | < 0.001 | 3.65 (2.20 to 6.04) | < 0.001 | 1.54 (0.73 to 3.23) | 0.257 |
| **Combined** | 8.25 (4.57 to 14.88) | < 0.001 | 9.64 (4.97 to 18.69) | < 0.001 | 3.14 (0.76 to 12.91) | 0.113 |
| * All information from all notes available were included as time-dependent variables. The model was adjusted by age, type of admission, and by the Australian and New Zealand (ANZ) Risk of Death (ANZROD) after log transformation | | | | | | |

| **Table S5 – Clinical Outcomes of Study Patients According to Behavioural Phenotype** | | | | | |
| --- | --- | --- | --- | --- | --- |
|  | **No Disturbance**  **(*n* = 1495)** | **Agitated**  **(*n* = 225)** | **Non-agitated**  **(*n* = 544)** | **Combined**  **(*n* = 667)** | ***p* value** |
| **Duration of ventilation, days*** | 0.0 (0.0 - 4.3) | 0.0 (0.0 - 15.7) | 0.0 (0.0 - 5.7) | 0.0 (0.0 - 29.3) | < 0.001 |
| **ICU length of stay, days** | 1.0 (0.6 - 1.8) | 1.8 (1.0 - 3.7) | 2.1 (1.3 - 3.7) | 4.9 (2.6 - 9.5) | < 0.001 |
| **Hospital length of stay, days** | 7.9 (4.9 - 14.0) | 9.5 (3.9 - 15.2) | 10.3 (6.5 - 18.3) | 15.9 (8.8 - 28.9) | < 0.001 |
| **ICU mortality - no. (%)** | 56 (3.8) | 21 (9.3) | 31 (5.7) | 47 (7.1) | < 0.001 |
| **Hospital mortality - no. (%)** | 76 (5.1) | 24 (10.7) | 52 (9.6) | 77 (11.6) | < 0.001 |
| **28-day mortality - no. (%)** | 71 (4.8) | 21 (9.4) | 45 (8.3) | 62 (9.4) | < 0.001 |
| Data as medians with interquartile range or number with percentage |  |  |  |  |  |
|  | | | | | |

| **Table S6 – Phenotype Transitions, Day 1 to Day 2** | | | | | | | |  |
| --- | --- | --- | --- | --- | --- | --- | --- | --- |
|  | | **Day 2** | | | | | |  |
| **Day 1** |  | **No Disturbance** | | **Agitated** | **Non-agitated** | **Combined** | **Total (Day 2)** |  |
|  | **No Disturbance** | - | | 27 (21.43) | 42 (33.33) | 57 (45.24) | 126 |  |
|  | **Agitated** | 16 (45.71) | | 9 (25.71) | 1 (2.86) | 9 (25.71) | 35 |  |
|  | **Non-agitated** | 33 (70.21) | | 0 (0) | 8 (17.02) | 6 (12.77) | 47 |  |
|  | **Combined** | 53 (59.55) | | 4 (4.49) | 4 (4.49) | 28 (31.46) | 89 |  |
|  | **Total (Day 1)** | 102 | | 40 | 55 | 100 | 297 |  |
| Data as total transitioning and percentage of total on Day 1 | | |  | | | | | |
|  |  | | | | | | |  |

Figure S1. Flowchart of Participation


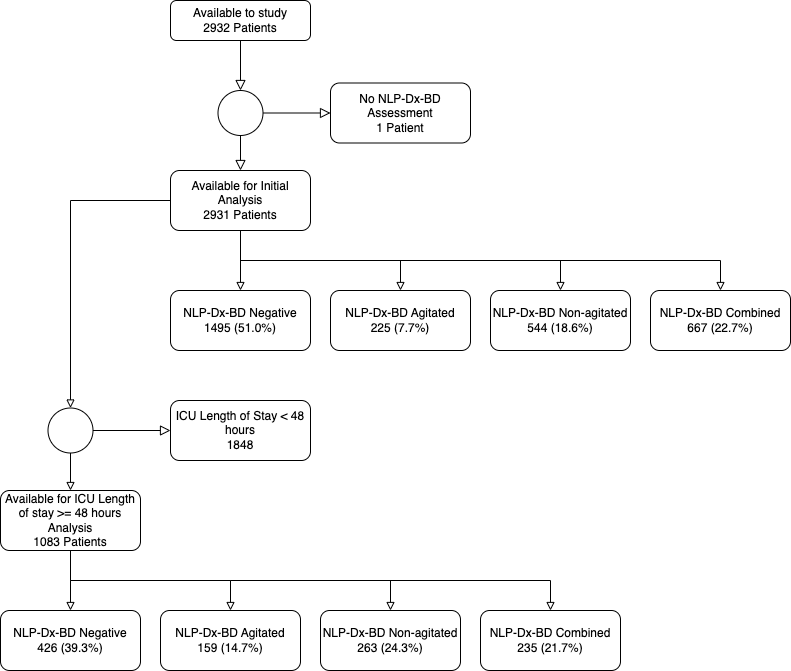


**Figure S2a. Agitated Behavioural State Word Cloud**


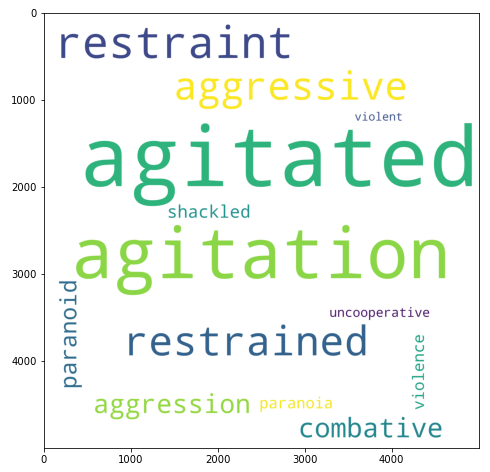


**Figure S2b: Non-agitated Behavioural State Word Cloud**


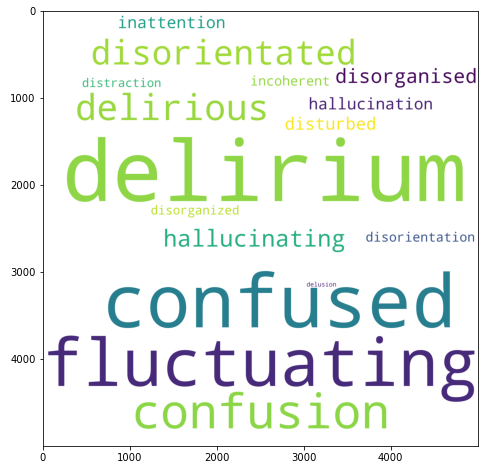


Figure S3. Days until Diagnosis


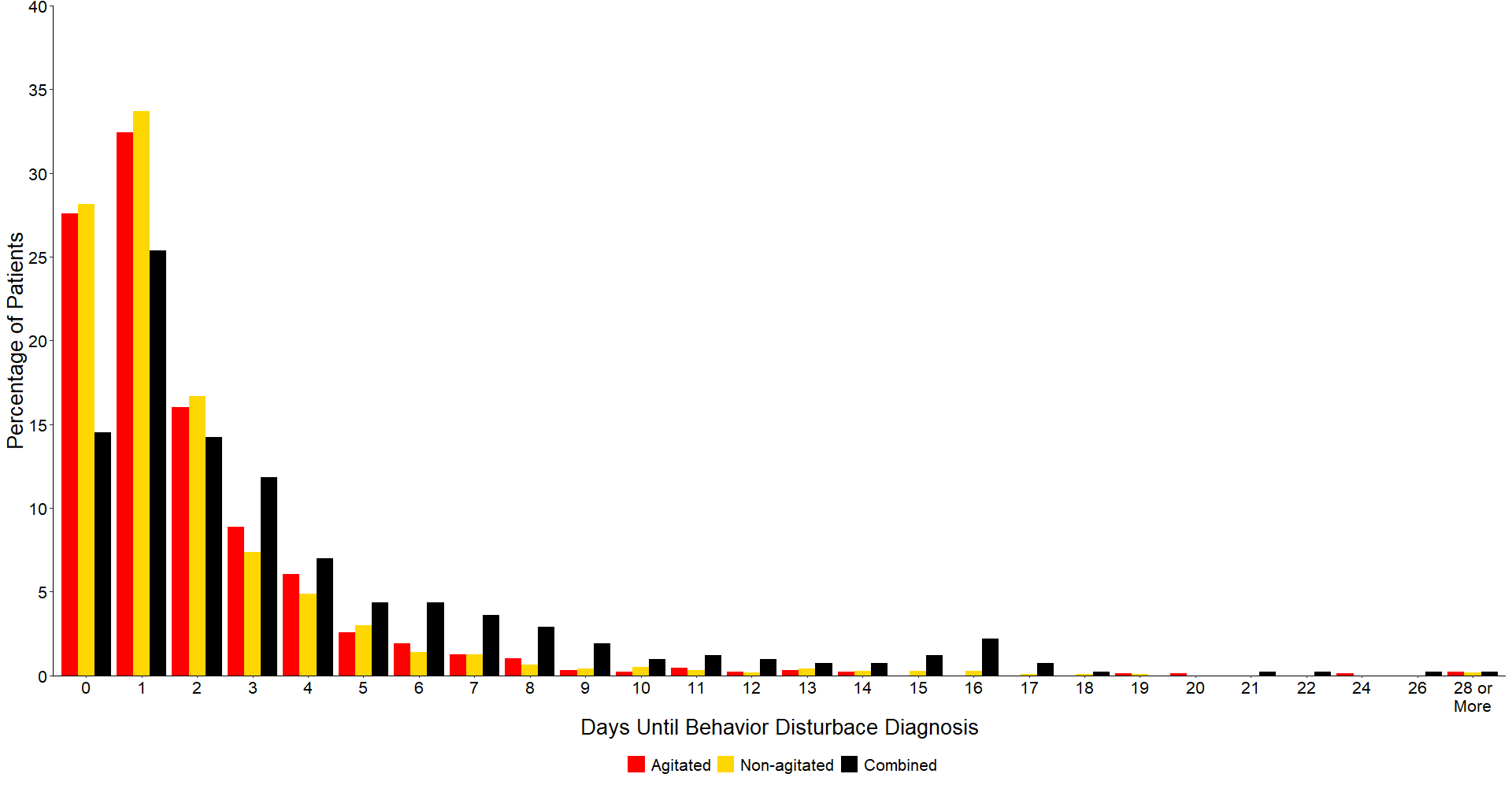

Supplement: Supplementary file 1 — Additional file 1: Online Supplement. [file 13054_2023_4695_MOESM1_ESM.docx]
